# The Value of Source Data Verification in a Cancer Clinical Trial

**DOI:** 10.1371/journal.pone.0051623

**Published:** 2012-12-12

**Authors:** Catrin Tudur Smith, Deborah D. Stocken, Janet Dunn, Trevor Cox, Paula Ghaneh, David Cunningham, John P. Neoptolemos

**Affiliations:** 1 Department of Biostatistics, University of Liverpool, Liverpool, United Kingdom; 2 Birmingham Cancer Research UK Clinical Trials Unit, University of Birmingham, Birmingham, United Kingdom; 3 Warwick Clinical Trials Unit, University of Warwick, Coventry, United Kingdom; 4 Cancer Research UK Liverpool Cancer Trials Unit, University of Liverpool, Liverpool, United Kingdom; 5 Department of Medicine, Royal Marsden Hospital, Sutton, Surrey, United Kingdom; Davidoff Center, Israel

## Abstract

**Background:**

Source data verification (SDV) is a resource intensive method of quality assurance frequently used in clinical trials. There is no empirical evidence to suggest that SDV would impact on comparative treatment effect results from a clinical trial.

**Methods:**

Data discrepancies and comparative treatment effects obtained following 100% SDV were compared to those based on data without SDV. Overall survival (OS) and Progression-free survival (PFS) were compared using Kaplan-Meier curves, log-rank tests and Cox models. Tumour response classifications and comparative treatment Odds Ratios (ORs) for the outcome objective response rate, and number of Serious Adverse Events (SAEs) were compared. OS estimates based on SDV data were compared against estimates obtained from centrally monitored data.

**Findings:**

Data discrepancies were identified between different monitoring procedures for the majority of variables examined, with some variation in discrepancy rates. There were no systematic patterns to discrepancies and their impact was negligible on OS, the primary outcome of the trial (HR (95% CI): 1.18(0.99 to 1.41), p = 0.064 with 100% SDV; 1.18(0.99 to 1.42), p = 0.068 without SDV; 1.18(0.99 to 1.40), p = 0.073 with central monitoring). Results were similar for PFS. More extreme discrepancies were found for the subjective outcome overall objective response (OR (95% CI): 1.67(1.04 to 2.68), p = 0.03 with 100% SDV; 2.45(1.49 to 4.04), p = 0.0003 without any SDV) which was mostly due to differing CT scans.

**Interpretation:**

Quality assurance methods used in clinical trials should be informed by empirical evidence. In this empirical comparison, SDV was expensive and identified random errors that made little impact on results and clinical conclusions of the trial. Central monitoring using an external data source was a more efficient approach for the primary outcome of OS. For the subjective outcome objective response, an independent blinded review committee and tracking system to monitor missing scan data could be more efficient than SDV.

## Introduction

The International Conference on Harmonisation (ICH) Good Clinical Practice (GCP) guideline [1] defines trial monitoring as “the act of overseeing the progress of a clinical trial and of ensuring that it is conducted, recorded and reported in accordance with the protocol, Standard Operating Procedures, GCP, and the applicable regulatory requirement(s)”. The primary aim of trial monitoring should be to assure patient safety and data quality. Whilst several approaches exist for monitoring clinical trials, they are generally classified under the headings of *on-site monitoring* and *central monitoring*.

On-site monitoring includes a range of different procedures for monitoring, each with a common theme that a member of the clinical trial team is required to visit one or more of the participating sites at one or more time points during the trial. Procedures performed during on-site visits are numerous and may include checking drug accountability, discussing recruitment and retention figures for the site, and review of screening logs and consent forms. One of the most common procedures undertaken during on-site monitoring is Source Data Verification (SDV), a procedure that is used to check that data recorded within the trial Case Report Form (CRF) match the primary source data which are contained in the relevant source document such as the medical record of the patient.

Central monitoring also includes a range of different procedures but is characterised by using centralised procedures instead of site visits. Procedures may include exploring accumulating data centrally to check for consistency over time and across different data items, statistical techniques to identify unusual data patterns within and across participating sites, and external validation of data items, such as through birth and death registries.

ICH GCP is not specific about the format of monitoring in clinical trials but suggests that “in general there is a need for on-site monitoring, before, during and after the trial and the use of central monitoring in conjunction with other procedures may be justified in exceptional circumstances” [1]. Unfortunately the guideline is frequently misinterpreted and clinical trials often routinely include on-site monitoring which can be inefficient, unnecessary and can result in the already limited resources being directed at quality assurance procedures that may be unimportant. Due to a growing concern about the effectiveness and efficiency of monitoring practices, and a lack of empirical evidence to determine which practices best achieve the goals of trial monitoring stated in ICH, the Clinical Trials Transformation Initiative (CTTI) project on effective and efficient monitoring [2] was initiated to identify best practices and provide sensible criteria to help sponsors select the most appropriate monitoring methods for a clinical trial. One recent output from this project is a survey of current practice which highlighted the varied approaches to monitoring and a lack of sufficient empirical evidence to determine which on-site monitoring practices lead to improved patient safety and data quality [3]. The CTTI project recommend building quality in to the trial design and focussing oversight on errors that are most likely to adversely affect trial quality, recognising that data elements vary in their impact on the safety of participants or on the reliability of trial results such that a single-minded focus on checking/ensuring accuracy of every data point is misguided [2].

Bakobaki et al [4] searched the literature recently and did not identify any trials that formally evaluated on-site monitoring techniques or directly compared multiple monitoring strategies against each other. They subsequently undertook a retrospective review of a selected sample of on-site monitoring reports from a large HIV prevention trial, concluding that 95% of the on-site monitoring findings reviewed could be identified using central monitoring strategies. Furthermore, Buyse et al [5], Baigent et al [6] and others have proposed that central monitoring is a more efficient approach for identification of fraud and anomalies of the data that are most likely to impact on results. Baigent et al [6] highlight an example from the Second European Stroke Prevention Study, in which fabricated data on 438 patients from one site was first detected by central monitoring methods which on-site monitoring had failed to identify.

The financial, human, and time resource required for on-site monitoring is greater than for central monitoring and this is likely to be a significant factor in the choice of approach used by commercial or non-commercial clinical trials. Results from a survey of Swedish pharmaceutical companies in 2005 suggested that fifty percent of the cost of GCP-related activities in phase III trials was due to SDV, with an estimated actual cost of SDV for a phase III program estimated as 90 million US Dollars [7]. In a different study, on-site monitoring was estimated to represent approximately 25 to 30% of costs in phase III cardiovascular clinical trials [8]. In 2000, Favalli *et al.* [9] evaluated the average cost per site visit in an oncology trial to be 1500 US Dollars not including the salaries lost through time taken from regular duties. Due to the high costs associated with site visits and SDV, and uncertainty about the effectiveness of these approaches, there is an urgent need to investigate the added value of on-site monitoring in terms of improving data quality and patient safety. The choice of monitoring practice should, as far as possible, be based on empirical evidence which is currently lacking in this important area.

Data are available from a parallel, open-label, multicentre (United Kingdom), phase III, superiority RCT comparing control with experimental treatments in patients with advanced cancer. The trial was designed and initiated before the introduction of the United Kingdom (UK) Clinical Regulations in May 2004 (Statutory Instrument 2004 Number 1031) and originally included a degree of central monitoring for missing and unusual data identified at each interim analysis and a planned blinded review of all response data. During the stages of final data collection, towards the end of the study, the Trial Management Group agreed to undertake 100% SDV through on-site visits to strengthen conclusions from the trial by assuring data quality. This paper describes an empirical comparison of the 100% source verified data against the corresponding unverified data and explores the value of SDV for this trial. We also explore the value of centralised procedures in this setting.

## Methods

Between May 2002 and January 2005 the trial recruited 533 patients from 75 secondary and tertiary care centres across the UK, all of which had research experience but variable in amount. Patient follow-up and death data were collected and entered on the trial database up until March 2006. During the conduct of the trial, all data were collected on paper CRFs and entered onto a central database. The prospectively planned quality assurance activities undertaken throughout included a programmed database designed to minimise input errors (e.g. drop down list rather than manual input, date checks in relation to dates of entry and treatments), planned interim analyses (3 interim analyses undertaken) which included statistical data cleaning of key variables, and blinded review of response data. This data set will be referred to as the *original data*.

After the trial had closed to recruitment but with some patients in active follow-up, a retrospective monitoring plan was developed to include 100% SDV of all important identified data items for all patients to verify that data in the CRF were consistent, complete and correct when compared with the source such as patient’s hospital notes. A small team of experienced monitors were employed to undertake the planned independent SDV activities in parallel to the trial itself between 2006 and 2007. All source verified data were re-entered onto a new database, independent to the original trial database, with manual and computer generated verification checks. This data set will be referred to as the *SDV data.*

Since the SDV was undertaken towards the end of the trial some of the events observed in the SDV data were due to having observed a longer patient follow-up compared to the original data. Therefore, to increase comparability and ensure as far as possible that any differences observed are due to SDV, a common ‘censoring date’, chosen as the last date of death recorded on the original database (8/3/06), was used across both data sets. Follow-up data from the visit prior to this censoring date were used where relevant in calculations and data recorded after this censoring date were ignored for the purpose of this empirical comparison. A sensitivity analysis ignoring this censoring date was also explored for the primary outcome.

In order to explore the value of SDV in this setting we assessed whether SDV uncovered data errors related to critical items, but more importantly whether these data errors affected the main trial results and related conclusions. Therefore, both data sets were compared in terms of baseline data, primary outcome (Overall Survival (OS)), and secondary outcomes (Progression Free Survival (PFS); Objective response; Serious Adverse Events (SAEs)) of the trial. The trial did also collect data for two patient reported outcomes but SDV has limited value for these outcomes as the source data are the original patient completed questionnaires which were routinely returned to the Clinical Trials Unit (CTU).

The following were calculated for each patient using both data sets:

- time from randomisation to death from any cause or last follow-up for those patients still alive at the common censoring date
- time from randomisation to progression or death from any cause, or last follow-up for those patients still alive and progression free at the common censoring date
- Response assessed in accordance with the World Health Organization (WHO) criteria for disease response (Response Evaluation Criteria in Solid Tumors (RECIST)) Guidelines [10] and reported as best achieved response with criteria determined as follows:
  - Complete response (CR): disappearance of all target lesions
  - Partial response (PR): At least a 30% decrease in the sum of the longest diameter of target lesions, taking as reference the baseline sum longest diameter
  - Stable disease (SD): Neither sufficient shrinkage to qualify for partial response nor sufficient increase to qualify for progressive disease, taking as reference the smallest sum longest diameter since the treatment started
  - Progressive disease (PD): At least a 20% increase in the sum of the longest diameter of target lesions, taking as reference the smallest sum longest diameter recorded since the treatment started or the appearance of one or more new lesions

The number of discrepancies identified are summarised for clinically relevant baseline characteristics that are typically reported in randomised controlled trials (RCTs) in this particular clinical setting. For time-to-event outcomes (OS and PFS), Kaplan Meier survival curves, log-rank analyses, and unadjusted and adjusted (adjusted for stratification factors at randomisation for OS only) Hazard Ratio (HR) estimates with 95% confidence intervals (CI) obtained from Cox regression models, were compared across data sets descriptively. For each dataset, overall response was compared across treatment groups using a chi-square test and by estimating the Odds Ratio (OR) and 95% CI. A simple comparison of number of SAEs recorded per patient in each dataset was undertaken. Differences in recording methods between datasets made more in-depth comparisons of SAEs difficult.

### Central Monitoring for Overall Survival

The Office for National Statistics (ONS) collects registration of birth and death data which can be made available for research studies through flagging, provided that the appropriate ethics approvals are in place. The use of independently collected birth and death data is a form of central monitoring and is useful to confirm the existence, date and cause of death for clinical trial participants.

ONS flagging was not prospectively planned for this study and so retrospective collection was necessary. A section 60 application was submitted to the patient information advisory group (PIAG) to gain approval to collect patient identifiers from participating sites. The multicentre research ethics committee (MREC) were notified, and a substantial amendment submitted to the Medicines and Healthcare products Regulatory Agency (MHRA). Following these approvals, the NHS number, name, and date of birth were obtained from participating sites and used for matching by the ONS. The paper copies of ONS death data were then entered onto a database by the trial team and verified through double data entry.

The ONS data provide a further source for the empirical comparison of the primary outcome OS. Time from randomisation to ONS date of death, or last follow-up for those patients still alive at the common censoring date, were calculated and compared to the original and SDV data using the methods described above for OS.

## Results

Data for all 533 randomised participants were verified against source data. Discrepancies in baseline characteristics were detected between the original and SDV data (Table 1). The percentage of patients with a discrepancy for each characteristic was generally low and equally distributed across treatment groups (Table 1) and participating sites (data not shown). In the original data, 4 patients were identified as ineligible following randomisation. Three patients had a pre-randomisation CT scan outside the permitted 30 day interval and one patient had a different cancer type to that listed as eligible in the protocol. However, the process of SDV failed to identify these four patients as ineligible which led to the discrepancy in Table 1. Other than this, there were no systematic patterns to the direction of discrepancies.

**Table 1:** Baseline discrepancies between SDV and original data.

BASELINE CHARACTERISTIC	DISCREPANCIES NUMBER OF PATIENTS (%)		
	CONTROL 266	EXPERIMENTAL 267	TOTAL 533
ELIGIBILITY CRITERIA*	2 (0.8)	2 (0.7)	4 (0.8)
STAGE	8 (3.0)	9 (3.4)	17 (3.2)
WHO PERFORMANCE STATUS	9 (3.4)	7 (2.6)	16 (3.0)
GENDER	2 (0.8)	1 (0.4)	3 (0.6)
DATE OF BIRTH	6 (2.3)	6 (2.2)	12 (2.3)
ETHNIC GROUP	1 (0.4)	6 (2.2)	7 (1.3)
DATE OF DIAGNOSIS	27 (10.2)	26 (9.7)	53 (9.9)

* Four ineligible patients were not identified as ineligible by SDV.

### Overall Survival

#### (i) Comparison of SDV against original data

**Figure 1:**
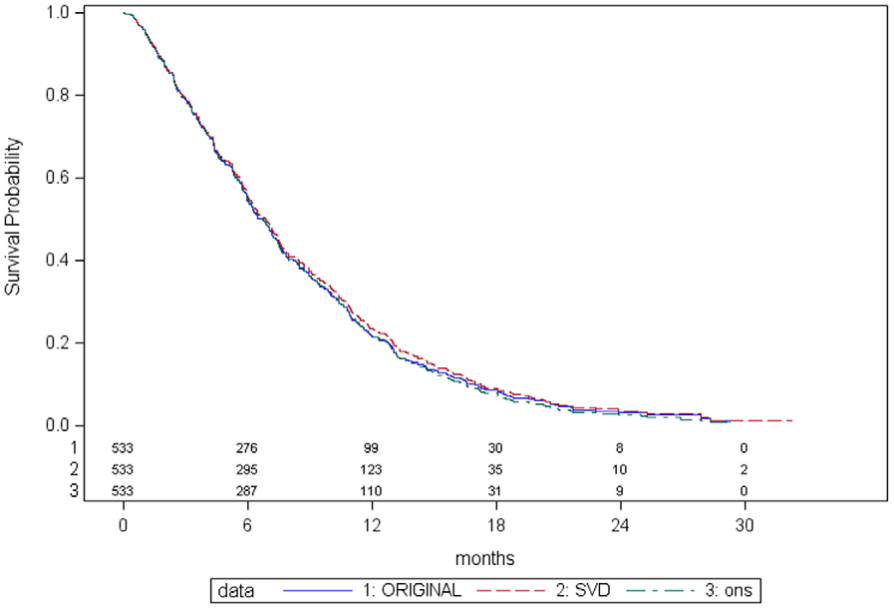
Kaplan Meier curves comparing SDV, original, and ONS data: Overall Survival.

A total of 13 (2.4%) participants had a discrepancy in date of death between the SDV and original data. The proportion, magnitude, direction and type of discrepancy in dates were similar with no systematic pattern across treatment groups (Table 2) or sites (data not shown), and suggest that transcription errors were the most likely explanation in the majority of cases. For a further 29 (5.4%) participants, the SDV process identified a date of death that had not been recorded in the original data which raised a discrepancy. The proportion of these discrepancies were also equally distributed across treatment groups [15] (5.6%) in control group and 14 (5.2%) in experimental group). All additional deaths identified through SDV occurred after the last date of follow-up recorded in the original dataset and were mostly deaths that occurred towards the end of the trial. The Kaplan Meier survival curve for overall survival (Figure 1) shows almost identical curves for the original and SDV data with a negligible effect on the treatment effectiveness analysis regardless of whether or not adjusted for *stage* and *performance status* (Table 3). Results were almost identical in a sensitivity analysis using all available SDV data regardless of the censoring date used in the empirical comparison.

**Table 2:** Date of death discrepancies between SDV and original data.

Control (n = 266)			Experimental (n = 267)		
Total discrepancies 5 (1.9%)			Total discrepancies 8 (3.0%)		
Original Date of death	SDV date of death	Days difference (SDV-original)	Original Date of death	SDV date of death	Days difference (SDV-original)
14/12/2002	17/12/2002	3	29/09/2002	24/09/2002	−5
15/12/2004	06/01/2005	22	03/10/2003	11/10/2003	8
07/09/2003	07/09/2004	366	26/01/2004	24/01/2004	−2
21/08/2004	21/09/2004	31	28/04/2004	26/04/2004	−2
25/11/2004	26/11/2004	1	19/12/2003	19/12/2004	366
			21/01/2004	02/01/2004	−19
			29/10/2003	28/10/2003	−1
			07/02/2005	08/02/2005	1

#### (ii) Comparison of central monitoring (ONS data) versus SDV

There were 53 (9.9%) discrepancies in date of death between ONS and SDV data. At the time of final analysis of the trial data ONS were unable to confirm a date of death for 5 of these patients. The SDV and original data agreed in four of these cases and date of death was also subsequently confirmed by site staff. However, for one of these patients that could not be confirmed as dead by ONS, original data recorded the patient as still alive whilst SDV data recorded this patient’s status as ‘still alive’ but also recorded a date of death (01/04/2005). Although likely that this patient had died by the time of analysis and thus should have been included in the ONS data records, we included the patient as a censored observation in these analyses. For one further patient, ONS identified a date of death which had not been recorded in either the SDV or the original data.

The Kaplan Meier survival curve (Figure 1) and unadjusted treatment effectiveness analysis (Table 3) using centrally monitored data are almost identical to the SDV and original data analyses.

**Table 3:** Treatment effectiveness analysis results – comparison of data sets.

	Original	SDV	Centrally monitored
***Overall Survival***			
HR* (95% CI)unadjusted	1.18 (0.99 to 1.42)	1.18 (0.99 to 1.41)	1.18 (0.99 to 1.40)
adjusted for stage and performance status	1.19 (0.99 to 1.43)	1.17 (0.98 to 1.40)	
Number of patients	533	533	533
Number of deaths	469	498^1^	499
Log-rank statistic	3.33	3.44	3.22
Log-rank p-value	0.068	0.064	0.073
***Progression Free Survival***			
HR* (95% CI)	1.29 (1.08 to 1.54)	1.30 (1.09 to 1.54)	
Number of patients	532^2^	532^3^	
Number of events	501	522	
Log-rank statistic	7.99	8.76	
Log-rank p-value	0.005	0.003	

* HR >1 indicates a benefit to experimental treatment.

1 Discrepancy in number of deaths between SDV and original data is due to the identification of 29 additional dates of death (all dates occurred before the censoring date used in this comparison) following source data verification. These patients were censored in the ‘original’ analysis using date of last follow-up.

2 Date of progression missing for one patient (id 113).

3 Date of progression is before date of randomisation for one patient (id 468).

### Progression Free Survival

**Figure 2:**
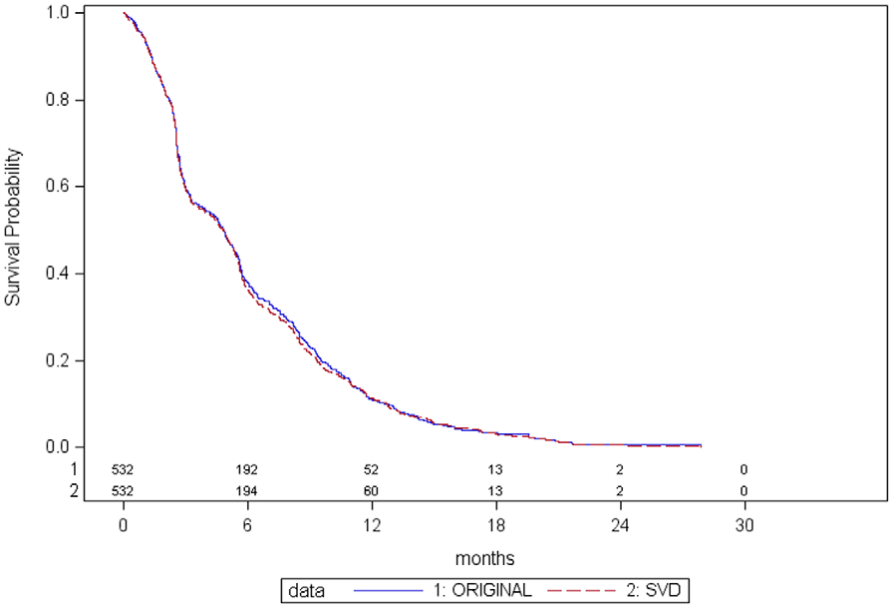
Kaplan Meier curves comparing SDV and original data: Progression Free Survival.

For the comparison between SDV and original data, there were a total of 132 patients (24.8%) with a discrepancy in the derived PFS time (median discrepancy 0.1 months, lower quartile −1.8 months, upper quartile 1.5 months, minimum −13.3 months, maximum 12.7 months). The percentage of discrepant observations are similar across treatment groups with no systematic pattern to the direction or magnitude of discrepancy. The Kaplan Meier survival curves for PFS (Figure 2) for the SDV and original data are again almost identical with a negligible effect on the treatment effectiveness analysis (Table 3).

### Response

RECIST response classifications are based on CT scan results undertaken at specified time points during the trial to assess change in tumour size from baseline. Across both the SDV ad original data there were a total of 620 CT scans but only 460 (74.2%) had been assigned a RECIST classification in both data sets. For these 460 scans, there was agreement in RECIST classification for 398 (86.5%) but disagreement for 62 (13.5%). The majority of these disagreements (58 (93.5%)) were due to a change in classification of one level up or one level down e.g. PD to SD, with the remaining 4 scans classified as PR in the original data set but classified as PD in the SDV data. A total of 160 scans were not common to both datasets; 125 scans which were identified through SDV and 35 scans that were missed during this process but which had been recorded in the original data (Table 4). Information is not available to explore the reason for these discrepancies. The SDV process is likely to have identified additional scans that were undertaken outside of the trial protocol 12-week schedule.

**Table 4:** RECIST response classification for each CT scan comparing SDV and original data (numbers relate to CT scans not patients).

		SDV classification					
		CR	PR	SD	PD	Missing*	Total
**Original classification**	**CR**	5	0	0	0	0	5
	**PR**	1	75	17	4	8	105
	**SD**	0	18	202	17	20	257
	**PD**	0	0	5	116	7	128
	**Missing** *	7	23	48	47	0	125
	**Total**	13	116	272	184	35	620

* No scan result available in the respective data set.

To explore how these scan level discrepancies translate to the patient level overall response analysis, the best achieved response across all scans was identified for each patient within each dataset. These response classifications (patient level rather than scan level) and treatment effectiveness analyses for this outcome were compared across treatment groups and dataset (Table 5). Although both datasets suggest a significantly better response rate for experimental treatment compared to control (lower part of Table 5), the original data provides a more extreme result (odds ratio 2.45) in favour of experimental treatment than the SDV data (odds ratio 1.67). This could suggest a potential bias in the original dataset as clinicians interpreting scan data were not blind to treatment allocation and may therefore be more likely to favour a better response classification for patients on the experimental treatment. However, during the trial, all scans were reviewed by a single clinician unaware of the treatment allocation (original classification). In addition, the suggestion of bias does not appear to be supported by the data as the percentage of patients who had a better response outcome in the SDV data compared to the original (7.5% on control, 7.6% on experimental), or a worse response outcome in the SDV compared to the original data (6.7% on control, 8.9% of patients on experimental) is similar across treatment groups. These data are based on 134 control and 158 experimental patients with an overall response classification available in both datasets (data not shown).

**Table 5:** Response analysis comparing SDV and original data (numbers relate to patients).

	Original		SDV	
	Control	Experimental	Control	Experimental
	(266)	(267)	(266)	(267)
**Best achieved response** n (%)				
CRPRSDPDCT scan not available	026 (9.8)77 (28.9)37 (13.9)126 (47.4)	4 (1.5)52 (19.5)71 (26.6)40 (15.0)100 (37.5)	1 (0.4)32 (12.0)78 (29.3)52 (19.5)103 (38.7)	8 (3.0)43 (16.1)79 (29.6)42 (15.7)95 (35.6)
**Overall Response (CR+PR)**				
OR* (95% CI)Chi-square test p-value	2.45 (1.49 to 4.04)0.0003		1.67 (1.04 to 2.68)0.03	

* OR >1 indicates a benefit to experimental treatment.

### Serious Adverse Events

Overall there were 53 patients (9.9%) with a discrepancy in the number of SAEs between datasets; 20 patients had 29 additional SAEs recorded in the original dataset and 33 patients had 36 additional SAEs recorded in the SDV data (Table 6). There are more discrepancies between datasets for patients on control (33 patients with 40 events) compared to experimental (20 patients with 25 events). This imbalance is not substantial but could suggest bias in the reporting of SAEs.

**Table 6:** Patients with discrepancy in number of Serious Adverse Events.

	Number of patients with discrepancy in SAEs		
	Control (266)	Experimental (267)	Total (533)
**Patients with additional SAEs recorded in original data**	**13**	**7**	**20**
* Patients with 1 additional event* * Patients with 2 additional events*	*7* *6*	*4* *3*	*11* *9*
**Patients with additional SAEs recorded in SDV data**	**20**	**13**	**33**
* Patients with 1 additional event* * Patients with 2 additional events*	*19* *1*	*11* *2*	*30* *3*
**Total patients**	**33**	**20**	**53**

### Estimated Costs

It is difficult to obtain the full costs of alternative monitoring approaches for a retrospective analysis such as this. In this particular example, the main additional financial costs of SDV would have been for monitors’ salaries and expenses incurred during the monitoring visits. There were 533 patients recruited from across 75 sites with an average of 7.1 patients per site. Assuming it would take an average of 2 hours per patient to undertake a complete SDV for the overall survival primary outcome, the process would have taken an estimated 1066 hours, equivalent to an estimated 30.5 working weeks (7 hours per day, 5 days per week). Assuming an average salary for a clinical trial monitor of £26,000 per annum (£31,306 annual gross cost), and an average of £100 per week in expenses, a conservative estimate of the cost of SDV for the primary outcome is £21,412. The cost of the alternative central monitoring process was estimated to be approximately £2,023 to include ONS costs (approximately £500) and data manager costs (£1523) based on 3 working weeks at salary of £22,000 per annum (£26,406 annual gross cost) to apply for section 60 permission, obtain the patient identifiers from site, submit to ONS (name, date of birth and NHS number where available – minimum data was name and date of birth), computerise and validate dates of death. Neither of these estimates have accounted for the time and financial resources required at each site which might reasonably be expected to be greater for the SDV process.

## Discussion

Results have been presented for an empirical comparison of SDV data against original trial data, and also against centrally monitored data for the primary outcome overall survival. The data used for this empirical comparison are quite unique as the comparison relates to a non-commercial clinical trial of an investigational medicinal product (CTIMP) for which 100% SDV was performed independently of the main trial. This trial was initiated prior to EU clinical trials directives and the current UK Clinical Trial requirements which now require GCP training of trial staff, Clinical Trial Authorisation and MHRA inspections of the trial documents as well as site files. Cancer clinical trials in the UK have also benefited from huge changes in research culture with the instigation of the National Cancer Research Network. The current culture of research governance and regulations which aim to safeguard the quality of clinical trials and safety of patients would make this particular empirical comparison difficult to repeat in future.

The comparison identified discrepancies between monitoring procedures for the majority of variables examined, with some variation in discrepancy rates. The potential for bias is greatest when errors are non-random with respect to treatment allocation [6]. In this example, the identified discrepancies for the baseline variables and the data required to construct the outcomes OS and PFS did not differ systematically across treatment groups or across sites, suggesting that random transcription errors were the most likely explanation for the majority of variables. For the two time-to-event outcomes the effect of these discrepancies on overall clinical results and conclusions was negligible.

An important, if not surprising finding of this work is that SDV does not necessarily provide error-free data. In this example, SDV failed to identify four patients that were classified as ineligible in the original data. Due to the age and retrospective nature of this data, we can only speculate that this was due to the monitors’ lack of clinical knowledge. In reality SDV is an iterative process and so it is possible that this discrepancy in eligible patients would have eventually been identified and resolved by the trial team. However, discrepancies in dates of death were also identified when SDV data were compared with centrally monitored data obtained from ONS. It is unlikely that the ONS data would contain errors but even if it did, these would be expected to occur completely randomly and be unrelated to the trial, outcome or treatment and would therefore provide unbiased data for estimation of the treatment effect. Together with the additional time and expense of SDV, estimated to be around £19,389, these findings suggest that the central monitoring procedure of using ONS data is the optimum approach for assuring the quality of primary outcome data for this trial.

The analysis of RECIST classification data highlighted important issues both in terms of the identification of additional scans, and in terms of interpretation of scan data. Discrepancies were most evident, and also had the most impact on results, for this subjective outcome. The number of additional scans identified through SDV were similarly distributed across treatment groups and they most likely reflect the continued clinical monitoring of patients at sites which were not requested as part of the trial protocol, or may not have always been fed back to the trials unit. Furthermore, given that there were discrepancies identified between SDV and the ONS date of death, and that SDV failed to identify 4 ineligible patients and 35 scans that were present in the original dataset, we cannot be certain that the SDV data is necessarily accurate for this outcome, particularly due to its subjective nature. It is possible that the monitor assessing the scan data may not have had the full medical information or knowledge required to make an accurate clinical assessment, a concern that has been raised in a previous study [11], as monitors may be less experienced and knowledgeable in the clinical area compared to investigators at sites. Alternatively, the clinicians/trial researchers who made the original assessment may have been biased in some way because their assessment was unblinded to the patient’s treatment allocation. However, a second review was undertaken by a blinded clinician, and the data we explored showed a similar distribution across treatment groups of the percentage of patients with an improved response classification between datasets. For this subjective outcome a robust tracking system for monitoring receipt of expected scans during the trial, and an independent endpoint review committee blinded to treatment allocation may have been the optimal method of quality assurance.

Monitoring approaches were difficult to compare in relation to SAE data due to the variation in recording and necessary use of open text fields. However, discrepancies in the number of SAEs per patient were identified between datasets. This is an important finding as trial investigators are required to report SAEs to protect patient safety, and a total of 65 additional SAEs were identified either in the original data that weren’t in the SDV data (29 additional events for 20 patients), or in the SDV data that weren’t in the original data (36 additional SAEs for 33 patients). Due to the retrospective nature of this comparison it was not possible to explore the reason for these discrepancies. It is worth noting that fewer discrepancies were identified in the experimental group, possibly due to clinicians’ vigilance identifying and reporting SAEs for an experimental treatment? Further work is required to assess the value of SDV for identifying unreported SAEs. However, it is unlikely that 100% SDV across all patients would be required. Alternative, risk-proportionate strategies, perhaps focussed on less experienced sites or those with differing SAE reporting characteristics compared to other sites, with provision of regular and clear training, may be more efficient.

SDV is just one of the procedures undertaken during on-site monitoring and the results presented here should be viewed with this in mind. There are potentially useful on-site procedures that have not been explored within this empirical comparison. For example, the PRIME process [12] used observation by peer reviewers to improve protocol adherence and train site staff, which increased trial performance and consistency. As further empirical research is undertaken, decisions regarding the optimal use of resources during on-site visits will more likely be evidence based and risk proportionate. Baigent et al [6] suggest that resources used for on-site monitoring could be redirected more usefully to increase sample size, a strategy that may have been particularly useful in this trial which was originally designed to have 80% power to detect a difference as statistically significant at the 5% significance level. Of course, this strategy may not be appropriate in all trial settings and the ethical implications, and potential added costs of recruiting additional patients would need to be considered thoroughly and balanced against the potential gains.

Regulatory agencies have recognised the need for clinical trial oversight approaches that appropriately account for differing levels of risk associated with each specific trial. The Medicines and Healthcare products Regulatory Agency (MHRA) recommend a risk proportionate approach based on work undertaken by the MRC/DH/MHRA Joint Project on Risk-adapted Approaches to the Management of Clinical Trials of Investigational Medicinal Products [13]. The US Food and Drug Administration (FDA) are currently developing guidance to assist sponsors of clinical investigations in developing risk-based monitoring strategies and plans for investigational studies of medical products. The CTTI project on Effective and Efficient Monitoring [2] have recently issued recommendations which include (i) the need to focus on areas of highest risk for generating errors that matter, (ii) prospectively measure error rates of important parameters, and (iii) tailor monitoring approach (e.g., site visits, central, statistical) to the trial design and key quality objectives. These recent developments are important advances in clinical trial monitoring research and reaffirm the need to move away from a one-size fits all approach of resource intensive and inefficient approaches to clinical trial monitoring.

To our knowledge the comparison presented in this paper is the first empirical comparison that has explored the impact of monitoring method on clinical outcomes and trial conclusions. A randomised comparison of on-site visits versus no on-site visits has been attempted and reported in the literature [11]. However, this trial was terminated early and results could only be used to evaluate the impact of on-site initiation visits on patient recruitment, patients’ follow-up time, quantity and quality of data submitted to the trial coordinating office, none of which were found to differ between monitoring approaches. The study could not evaluate the impact of repeated on-site visits on clinical outcomes. Two further relevant studies are underway. The ADAMON project [14] is a German led cluster randomised study involving twelve clinical trials that randomise sites within each trial to a risk-adapted approach, or to an intensive monitoring strategy with frequent visits and 100% source data verification. The OPTIMON project [15] is a French led initiative comparing intensive monitoring that includes 100% SDV against an optimized risk-based monitoring approach. Results from both studies are expected in the next few years and will contribute important information, along with results from our study, such that future recommendations regarding monitoring practices may be more appropriately evidence-based.

### Conclusions

The value of the resource intensive source data verification needs to be established. In this example from cancer the process was time consuming, expensive, not necessarily error-free, and the resulting discrepancies identified made no impact on the main conclusions of the study. Source data verification did identify additional CT scan data which did impact upon the analysis of a secondary outcome of overall response. However, further empirical evidence is required to establish its value in this setting as it is likely that other more efficient methods such as effective tracking systems for missing scan data and independent blinded review of CT scans would be sufficient.

In conjunction with a thorough risk-proportionate monitoring system for the trial, one approach, if ethically reasonable, to safeguard against the effect of random errors might be to inflate the target sample size as is often done to account for potential missing outcome data.

### Strengths and Weaknesses

The example presented relates to an academic led, Cancer Research UK funded trial with short duration and minimal number lost to follow-up since patients with advanced cancer tend to stay in follow-up. The trial did not include a per-patient payment for entering patients and there was no obvious incentive for fraud. The conclusions drawn may not necessarily apply to other clinical settings or to commercially funded trials which often attract significant payments to investigators for entering patients. However, as long as the potential for errors that are not random in relation to treatment allocation would not be expected to differ then the results from this empirical comparison should be generaliseable to other settings.

As the trial was conducted prior to the 2004 clinical trial regulations, monitoring practice in CTIMPs may have improved and the empirical comparison presented here may therefore represent a worst case scenario which in some respects is a more informative comparison. As the retrospective SDV was undertaken towards the end of the trial during which some patients were still in active follow-up, it is possible that the introduction of this additional trial process may have changed trial conduct. As this empirical comparison was not prospectively planned, insufficient information is available to thoroughly explore and provide explanations for identified discrepancies. Further confirmatory empirical studies of this nature are required.
